# Early Detection of Severe Functional Impairment Among Adolescents With Major Depression Using Logistic Classifier

**DOI:** 10.3389/fpubh.2020.622007

**Published:** 2021-01-26

**Authors:** I.-Ming Chiu, Wenhua Lu, Fangming Tian, Daniel Hart

**Affiliations:** ^1^Department of Economics, Rutgers University, Camden, NJ, United States; ^2^Department of Community Health and Social Medicine, School of Medicine, City University of New York, New York, NY, United States; ^3^Department of Psychology, Rutgers University, Camden, NJ, United States

**Keywords:** major depressive episode with severe impairment, National Survey on Drug Use and Health, recall/accuracy rate, machine learning, logistic regression model/classifier

## Abstract

Machine learning is about finding patterns and making predictions from raw data. In this study, we aimed to achieve two goals by utilizing the modern logistic regression model as a statistical tool and classifier. First, we analyzed the associations between Major Depressive Episode with Severe Impairment (MDESI) in adolescents with a list of broadly defined sociodemographic characteristics. Using findings from the logistic model, the second and ultimate goal was to identify the potential MDESI cases using a logistic model as a classifier (i.e., a predictive mechanism). Data on adolescents aged 12–17 years who participated in the National Survey on Drug Use and Health (NSDUH), 2011–2017, were pooled and analyzed. The logistic regression model revealed that compared with males and adolescents aged 12-13, females and those in the age groups of 14-15 and 16-17 had higher risk of MDESI. Blacks and Asians had lower risk of MDESI than Whites. Living in single-parent household, having less authoritative parents, having negative school experiences further increased adolescents' risk of having MDESI. The predictive model successfully identified 66% of the MDESI cases (recall rate) and accurately identified 72% of the MDESI and MDESI-free cases (accuracy rate) in the training data set. The rates of both recall and accuracy remained about the same (66 and 72%) using the test data. Results from this study confirmed that the logistic model, when used as a classifier, can identify potential cases of MDESI in adolescents with acceptable recall and reasonable accuracy rates. The algorithmic identification of adolescents at risk for depression may improve prevention and intervention.

## Introduction

Major depression is one of the most common mental disorders in adolescents. It is characterized by persistent feelings of emptiness, sadness, and frustration in teenagers (1, 2). Globally, depression is the leading cause of disability-adjusted life years lost in adolescents aged 10 to 19 (3). In the United States, suicide is the third major cause of death among adolescents ages 10–19, and depression is the leading risk factor (4). Numerous studies have linked adolescence-onset depression with adverse life outcomes such as academic failure, self-injuries, risky sexual behavior, and substance abuse in late adolescence, unemployment, crime, and deteriorating quality of life in adulthood, and life-long morbidity (1, 5, 6).

Depression also impairs financial progress in adulthood. Research indicates that depression in childhood and adolescence increases the use of healthcare services, school services, as well as social services (7, 8). If left untreated, adolescent depression can lead to lifetime economic crises through heavier use of resources for treatment, employment difficulty, and lost production due to work absence or early retirement. To reduce these severe health, social, and economic consequences, it is critical to identify adolescents with depression in early stage and provide them with adequate medical treatments. Many correlates of depression have been identified, including age, gender, family composition, and parenting style (9, 10). Most existing research in this area, however, has focused on identifying the direction and strength of such associations using traditional regression models and searching for potentially causal risk factors in the onset of depression rather than seeking to develop statistical models to predict those at risk to develop depression.

In recent years, researchers and healthcare providers have recognized the value of developing predictive models to identify those who are prone to developing or having an undiagnosed mental health disorder. A few recent studies (11, 12) have used machine learning to examine the predictors and levels of depressive symptoms, but none has attempted to build a predictive model to identify adolescents who are at higher risk for major depression, especially those with depression-related severe functional impairment that need more costly intensive care (13–15). This study aims to fill out this critical research gap by building a predictive model using machine leaning techniques to detect severe functional impairment among adolescents with depression. Specifically, we aim to (1) determine the direction and strength of association between sociodemographic characteristics and severe functional impairment in adolescents with depression using logistic regression, and (2) based on findings from the logistic regression in the training data, construct a predictive model for severe functioning impairment among adolescents with major depression.

## Materials and Methods

### Data

Pooled data for adolescents aged 12 to 17 from the National Survey on Drug Use and Health (NSDUH) 2011–2017 were used for this study. The NSDUH is an annual cross-sectional survey sponsored by the Substance Abuse and Mental Health Services Administration (SAMHSA) of the US Department of Health and Human Services. By using a stratified multistate area probability sampling method, the NSDUH survey provides nationally representative data for the civilian, non-institutionalized population aged 12 or older from all 50 states and the District of Columbia in the U.S. The survey is administered in English and Spanish, and interviews are conducted using computer-assisted interviewing (16). The NSDUH has been tested for validity and reliability and has weighted percent agreements ranging from 70.3 to 99.0% on most variables included in this study. In the NUSDH surveys 2011–2017, the weighted response rates for adolescents range from 75.07 to 85.0%. Detailed information about the NSDUH data collection and manipulation procedures can be found in the web site of the SAMHSA (17). The Institutional Review Board at RTI International approved the NSDUH data collection protocol (18).

### Measurement

In the NSDUH, major depression was measured using a structured interview based on the fourth edition of the Diagnostic and Statistical Manual of Mental Disorders (DSM-IV). Adolescents were classified as having a 12-month major depressive episode (MDE) if they had either depressed mood or loss of interest or pleasure in daily activities for 2 weeks or longer in the past 12 months, while also experiencing four or more other symptoms that reflect a change in functioning, such as problems with sleep, eating, energy, concentration, and self-worth.

Adolescents with 12-month MDE were further asked questions from the Sheehan Disability Scale to measure the level of MDE-related functional impairment in four major life activities or role domains, that is, chores at home, school, or work; close relationships with family; and social life. Ratings above 7 on a 0-10 visual analog scale with categories of “no interference” (0), “mild” (1–3), “moderate” (4–6), “severe” (7–9), and “very severe” (10) were considered severe impairment. For this study, we focused on adolescents with depression who were also diagnosed as having MDE-related severe impairment (MDESI), because they are in the most severe depressive state and in urgent need of early detection.

Sociodemographic variables examined in this study included adolescents' age group (12-13, 14-15, and 16-17 years), gender (male and female), race/ethnicity (white, Hispanic, non-Hispanic black, Asian/Native Hawaiian or other Pacific Islanders (NHPIs), and other), insurance coverage (uninsured or insured), annual household income (<$20,000, $20,000-$49,999, $50,000-$74,999, and ≥$75,000), and family structure (whether adolescents had a father, a mother, or any siblings under age 18 in the household).

In the NSDUH, adolescents were asked seven questions related to the extent of support, oversight, and control that they perceived their parents provided or exercised over them in the past 12 months, e.g., checking on whether they had done their homework, providing help with their homework when they needed it, and letting them know when they had done a good job. Response options included “always,” “sometimes,” “seldom,” and “never.” the overall scores were dichotomized based on the median split into two categories to indicate high and low levels of authoritative parenting.

**Figure 1:**
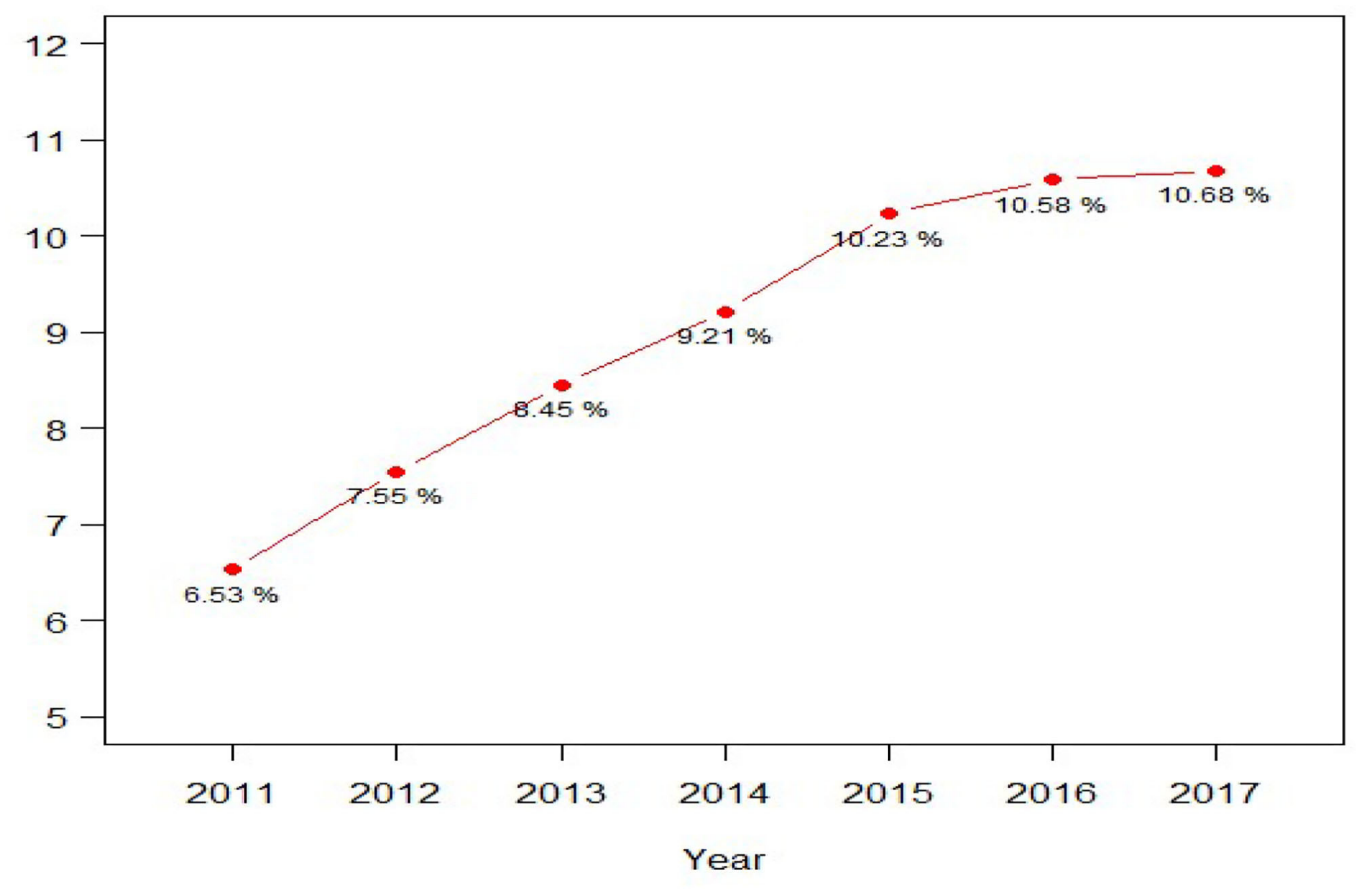
Prevalence of major depression-related severe impairment among adolescents 12 to 17 years old in the National Survey of Drug Use and Health (2011-2017) (*N* = 92,840).

Adolescents were also asked to rate six aspects of their school experiences in the past 12 months on a 4-item Likert scale, such as their overall feeling about going to school (1 = liked going to school a lot, 4 = hated going to school), how often their teachers at school let them know when they were doing a good job with their school work (1 = always, 4 = never), and their grades for the last semester or grading period their completed (1 = “A+”, “A”, or “A-”, 4 = “D”. For the study, adolescents' total scores on the 6 questions were dichotomized by the median split into positive vs. negative school experiences. Table 1 lists sample characteristics of the study. The annual proportions of MDESI cases among all adolescents are presented in Figure 1 over the 2011–2017 period.

**Table 1:** Sociodemographic characteristics of adolescents 12 to 17 years old in the National Survey of Drug Use and Health (2011–2017) (*N* = 92,840).

**Sociodemographic characteristic**	**Percentage**
**Gender**	
Male	52.8%
Female	47.2%
**Age**	
12–13	31.5%
14–15	34.2%
16–17	34.3%
**Race/Ethnicity**	
White	56.9%
Hispanic	19.7%
Black	12.9%
Asian/NHPIs	4.1%
Other	6.5%
**Insurance**	
Yes	95.4%
No	4.6%
**Household income**	
<20,000	16.5%
20,000-49,999	29.3%
50,000-74,999	16.5%
>75,000	37.4%
**Father in household**	
Yes	72.9%
No	27.1%
**Mother in household**	
Yes	91.9%
No	8.1%
**Siblings under 18 in household**	
Yes	69.0%
No	31.0%
**Authoritative parenting**	
High	86.3%
Low	13.7%
**School experience**	
Positive	83.0%
Negative	17.0%

### Data Analysis

The logistic regression model is a popular supervised machine learning tool. The word “supervised” indicates there is a mapping from a group of “features” Xs to a “target” variable Y. The concept is like when we use the term “independent” variables to describe Xs and “dependent” variable to describe Y in the regression models. Additionally, the term “classification” is used when Y is a categorical variable, and “regression” is used when Y is a quantitative variable. The standard procedure to apply any supervised learning method is to divide data into “training” and “test” datasets randomly. The split proportion is subject to data size; usually four to one (80% of training and 20% of test) or three to one (75% of training and 25% of test) split is used. Once the split is done, the logistic regression analysis is implemented using the training data and its results are used to build a classifier to differentiate diseased cases from disease-free cases. The performance of a classifier can be judged by criteria such as accuracy, recall, precision, and F1-score (19, 20). The criteria used in this study are addressed later in this section.

This data analysis and model performance evaluation process are conducted repeatedly until model improvement plateaus. While the initial process is done manually, the aim is to automate the process, converting the iterative process into a programming algorithm or “Machine Learning.” One recurrent challenge in machine learning is to avoid overfitting a model, which can reduce the value of the model for working with data not used in model development. The “overfitting” concept can be understood by using the goodness of fit measurement, *R*-squared value (*R*^2^), in the linear regression model. Overfitting is referred to as having a high *R*^2^-value of a model using the training dataset while obtaining a relatively low *R*^2^-value when the same model is applied using the test data. In other words, an overfitting model fits the training dataset too well but has poor fit with new datasets (19, 20).

In the following session, we briefly address the logistic model and explain how it can be used as a classifier to detect adolescents with MDESI. The logistic model can be represented using the following equation:

(1) $$\begin{array}{l} \text{E}(\text{Y}|\text{X})=\text{P}(\text{Y}=1|\text{X})=\frac{\exp(\text{X}*\beta )}{1+\exp(\text{X}*\beta )};\\ \text{ exp}:\text{exponential}\;\text{function}. \end{array}$$

Where Y is a binary variable that represents two states, MDESI and MDESI-free, and can be recoded using numeric values one and zero. X is a column of vector that represents the sociodemographic factors, and β is the associated coefficient vector. The conditional mean of Y given X, E(Y|X), is equivalent to the probability that Y is having MDESI, P(Y=1|X), which can be linked to a logistic function. The purpose of using the logistic function is to transform the infinite range of linear predictor values in X^*^β to a confined interval between zero and one, an interval that represents the probability of having MDESI. The maximum likelihood estimator is used to uncover the unknown coefficient vector β. In practice, the results are often presented using the following log of odds equation, where β_i_ can be interpreted as the effect of one-unit change in X_i_ on log of odds. Alternatively, we may take the exponentiation of β_i_, and this value can be explained as an odds ratio of comparing the relative risk of having depression in a category to its reference category.

(2) $$\text{log}(\frac{\text{P}}{1-\text{P}})=\text{X}*\beta$$

(3) $$\frac{\text{P}}{1-\text{P}}(\text{Odds})\text{​}=\exp(\text{X}*\beta ){\text{ore}}^{\text{X}*\beta };\text{exp}:\text{exponentialfunction}.\;$$

Once the estimation of β ($\hat{\beta }$) is found, the values of X^*^ $\hat{\beta }$ can be used as an input in Equation (4) in order to find the predicted probability $\hat{\text{P}}$ (a.k.a. score).

(4) $$\hat{\text{P}}(Y=1|X)=\frac{\exp(\text{X}*\hat{\beta })}{1+\exp(\text{X}*\hat{\beta })}$$

The key to building a successful classifier or predictive model hinges on the choice of θ, a threshold value used to categorize (predict) the cases into MDESI or MDESI-free states. By combining the true MDESI with MDESI-free cases, we can form a 2 by 2 confusion matrix below.

**Table:** 

**Confusion Matrix**		
Predicted\Actual	0	1
0	TN	FN
1	FP	TP

TP: True Positive (the predicted MDESI cases are indeed cases with MDESI)
TN: True Negative (the predicted MDESI-free cases are indeed cases without MDESI)
FP: False Positive (the predicted MDESI cases are actually cases without MDESI)
FN: False Negative (the predicted MDESI-free cases are actually cases with MDESI)
Accuracy = $\frac{\text{TP }+\text{ TN}}{\text{TP }+\text{ FN }+\text{ TN }+\text{ FP}}$ (the proportion of correctly identified cases in the entire sample)

Recall = $\frac{\text{TP}}{\text{TP }+\text{ FN}}$ (the proportion of correctly identified cases in the total of actual MDESI cases).

Each cell in the confusion matrix represents whether the state of predicted cases matches the state of actual cases, including true positives (TP), true negatives (TN), false positives (FP), and false negatives (FN). To measure the performance of a predictive model, we adopt two metrics, accuracy rate and recall rate. When the data are balanced with approximately equal number of MDESI and MDESI-free cases, accuracy rate alone can be used as a performance measurement. However, this measurement can be misleading if the data is imbalanced. Among 92,840 participants in this study, the MDESI cases only accounts for 8.84% of the sample. Therefore, a high accuracy rate can be attributed mostly by correctly identified MDESI-free cases, because they represent 91.16% of the sample. To overcome this problem, we take another performance metric, recall rate, into consideration. Recall rate is also called sensitivity. Our strategy is to choose an optimal threshold value by maximizing the recall rate while maintaining the highest possible accuracy rate. There are different methods to choose an optimal threshold value under the issue of imbalanced data. Our strategy is subjective, since the true cost of false identifications (FN and FP) is unknown.

In practice, 70–80% of the data is often used for model training purposes with the remaining data reserved for testing the model (21). We adopted this strategy and applied a three to one split to our sample; 75% of the observations (69,630 cases) was used for estimation purpose, and the rest 25% of observations (21,230 cases) were used to examine the validity of the predictive model. All the analysis and computations were done using the R software (R 4.0.2) (22).

## Results

The empirical results of the logistic regression model are reported in Table 2. The adjusted odds ratio (AOR) can be used to examine the relative risk of a group of each demographic variable in comparison to its reference group. For example, in regard to gender, the risk of having MDESI (4.25 to 1) was higher in girls than in boys (the reference group) (AOR = 4.25, *p* < 0.01). Compared to adolescents in the 12-13 age group, those in the 14-15 and 16-17 age group were more likely to have MDESI (AOR = 1.92, *p* < 0.01; AOR = 2.24, *p* < 0.05). Further, compared to Whites, lower risks of having MDESI were observed among Blacks (AOR = 0.70, *p* < 0.01) and Asian/NHPIs (AOR = 0.82, *p* < 0.01). As to family related factors and school experiences, living in a single-father or single-mother households significantly increased adolescents' chance of having MDESI (AOR = 1.14, *p* < 0.01; AOR = 1.12, *p* < 0.01). Having less authoritative parents and bad school experiences also increased the likelihood that adolescents have MDESI (AOR = 2.37, *p* < 0.01; AOR = 2.98, *p* < 0.01). Among those significant demographic factors, gender has the largest effect size, followed by negative school experiences. Lastly, no significant associations were noted between MDESI with either household income or adolescent insurance status.

**Table 2:** Logistic regression results.

	**Estimates**	**95% CI**	
**Variables**	**(AOR)**	**(Lower bound)**	**(Upper bound)**
(Intercept)	0.0000^***^	0.0000	0.0000
**Year**	1.0978^***^	1.0828	1.1129
**Gender (ref: boy)**	4.2529^***^	3.9972	4.5275
**Age (ref: Age 12–13)**			
Age 14–15	1.9236^***^	1.7778	2.0826
Age 16–17	2.238^***^	2.0702	2.4209
**Race (ref: White)**			
Hispanic	1.0124	0.9392	1.0907
Black	0.7021^***^	0.6362	0.7737
Asian/NHPIs	0.8174^***^	0.7016	0.9478
Other	1.1043^*^	0.9892	1.2305
**Insurance (ref: yes)**	0.9084	0.794	1.0357
**Income (ref: <$20,000)**			
$20,000–49,999	1.0197	0.9366	1.1106
$50,000–74,999	1.0437	0.9442	1.1537
$75,000 or more	0.9542	0.8698	1.0472
**Father in household (ref: yes)**	1.1436^***^	1.0693	1.2228
**Mother in household (ref: yes)**	1.1155^**^	1.0122	1.2275
**Siblings in household (ref: yes)**	1.0569^*^	0.9959	1.1215
**Authoritative parenting (ref: high)**	2.3742^***^	2.2253	2.5323
**School experience (ref: positive)**	2.9762^***^	2.8009	3.1621

* *p < 0.1*;

** *p < 0.05*;

*** *p < 0.01 AOR: adjusted odds ratio. All variables listed were included in the logistic model. Null deviance: 41924 on 69629 degrees of freedom; Residual deviance: 35,961 on 69,612 degrees of freedom AIC: 35,997; McFadden's Pseudo R^2^: 14.22%*.

**Figure 2:**
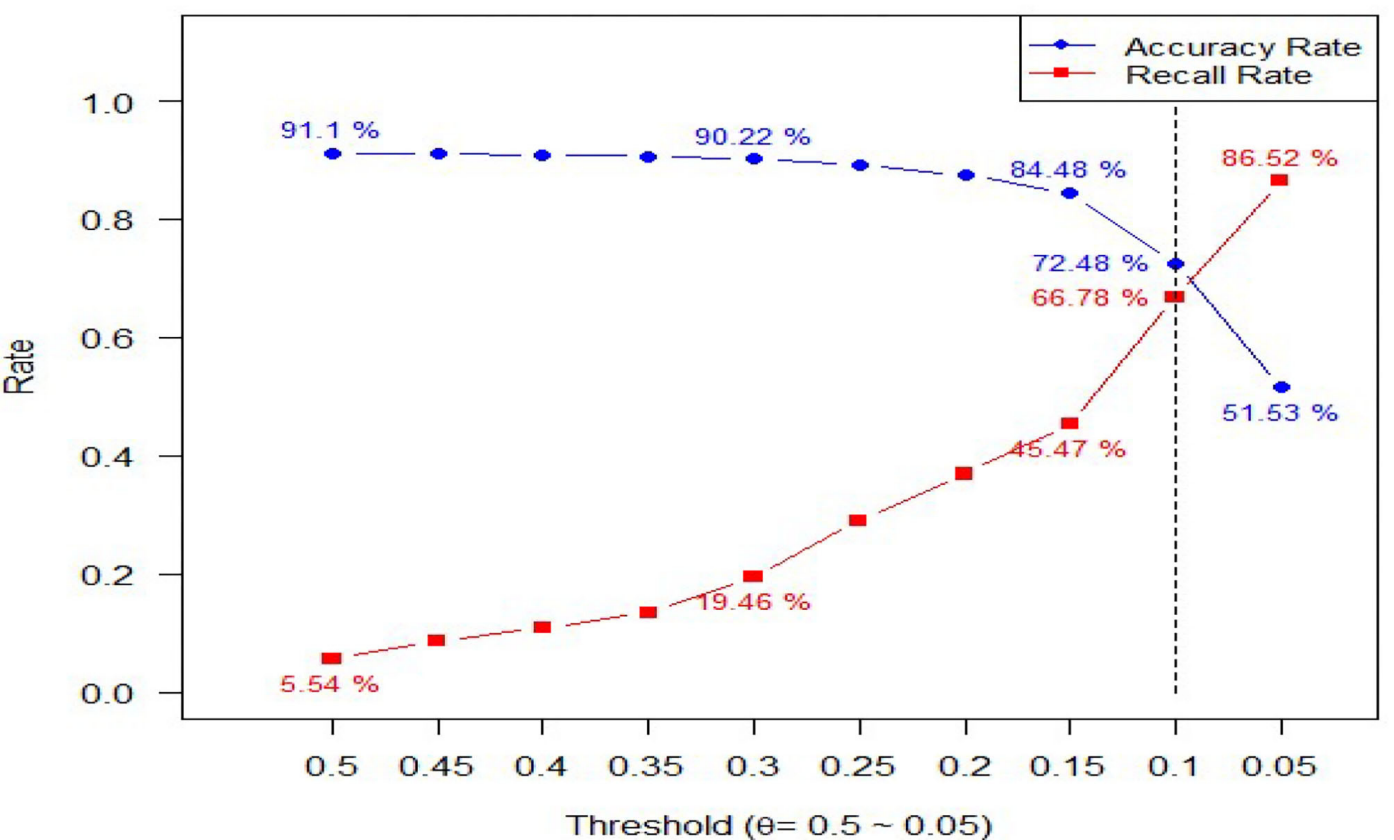
Criteria to choose optimal threshold value using Accuracy and Recall.

As explained in the Methods subsection, we searched for an optimal threshold value by maximizing the recall rate while maintaining the highest accuracy rate. Figure 2 illustrates the selection criteria. Both accuracy and recall rates were plotted using threshold values ranging from 0.5 to 0.05 (a descending order on horizontal axis). As shown in Figure 2, as threshold value decreased, the recall rate got higher while the accuracy rate got lower. Using both recall and accuracy rates as performance metrics, the optimal value θ was selected at 0.1, and the corresponding line segment was also plotted in Figure 2. Accordingly, 66.78% of recall rate and 72.48% of accuracy rate were achieved and the corresponding confusion matrix was reported in Table 3A. To avoid the overfitting problem, both accuracy and recall rates were computed using the test data. As reported in Table 3B, a recall rate of 65.77% and an accuracy rate of 72.31% were obtained. Both rates were close in training and test data, indicating no overfitting issue in the predictive model. Furthermore, a four-hold cross-validation method was applied to examine the general performance of the predictive model, and the corresponding recall and accuracy rates were all about the same.

**Table 3:** Confusion matrix.

**(A)**		
θ = 0.1 (training)		
**Predict\Actual**	0 (No MDESI)	1 (MDESI)
0 (No MDESI)	46316 (TN)	2067 (FN)
1 (MDESI)	17092 (FP)	4155 (TP)
Recall rate = 66.78%; Accuracy rate = 72.48%		
**(B)**		
θ = 0.1 (test)		
**Predict\Actual**	0 (No MDESI)	1 (MDESI)
0 (No MDESI)	15481 (TN)	678 (FN)
1 (MDESI)	5748 (FP)	1303 (TP)
Recall rate = 65.77%; Accuracy rate = 72.31%		
**(C)**		
θ = 0.5 (training)		
**Predict\Actual**	0 (No MDESI)	1 (MDESI)
0 (No MDESI)	63091 (TN)	5877 (FN)
1 (MDESI)	317 (FP)	345 (TP)
Recall rate = 5.54%; Accuracy rate = 91.10%		

## Discussion

Using data from NSDUH, this study utilized a modern supervised learning method—the logistic classifier—to identify/predict adolescents with MDESI. The logistic regression model was first applied to uncover the direction and strength of associations between MDESI and a list of sociodemographic factors. The empirical findings are mostly consistent with past findings in relevant researches (10). For example, compared to boys, girls had a much higher risk of MDESI. In terms of race/ethnicity, lower risks were observed in Blacks and Asian/NHPIs than Whites. Findings in this study also revealed that family structure, parenting style, and school experiences were associated with MDESI and can be used to identify potential MDESI cases.

**Figure 3:**
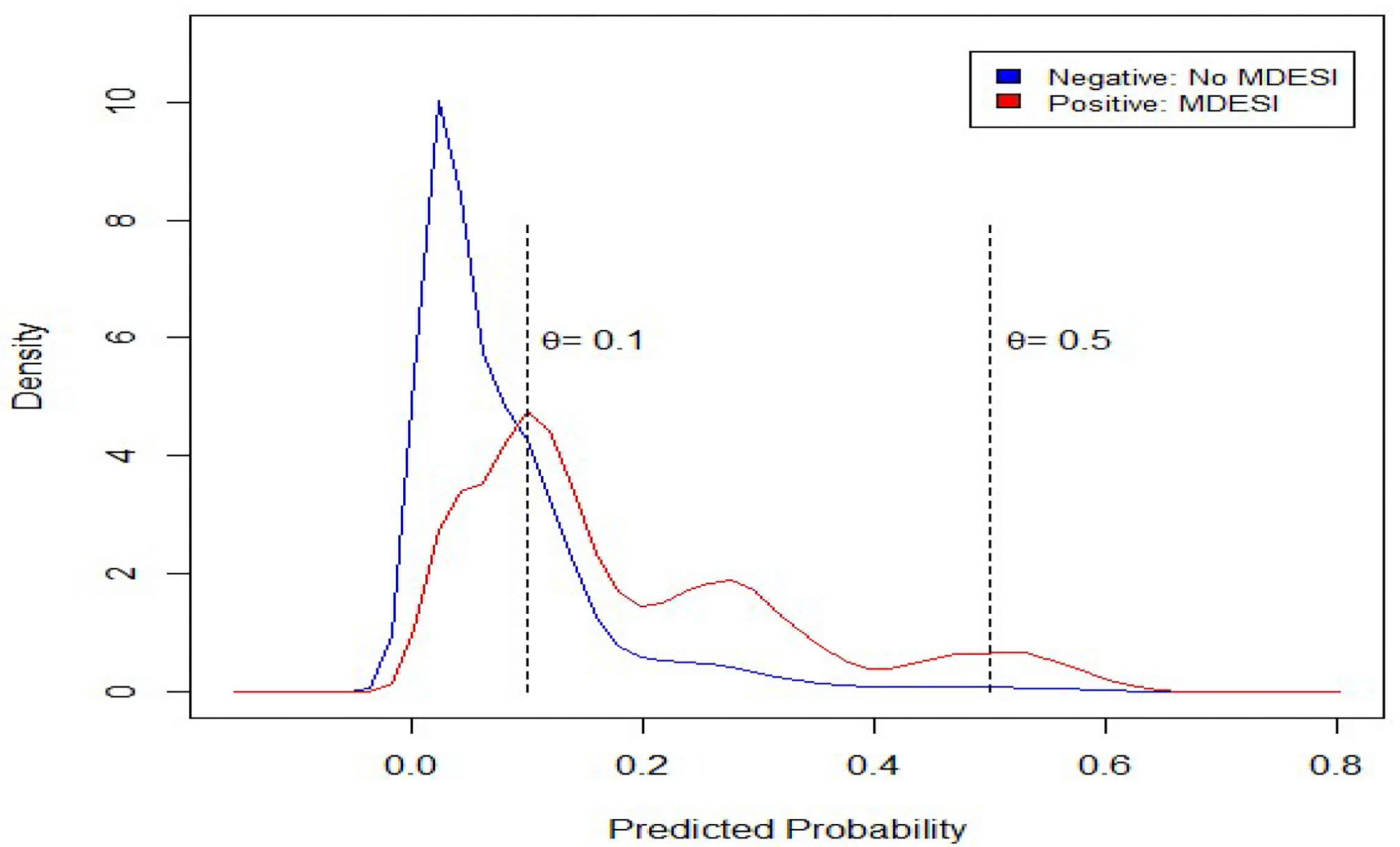
Density plot for predicted probability obtained from the logistic model.

Our ultimate goal for this study is to construct a classifier to identify cases with MDESI. Therefore, the predicted probability must be computed using Equation (4) listed in the Methods section. The densities of the predicted probability are plotted in Figure 3, in which red and blue colors are used to differentiate MDESI and MDESI-free cases. Both densities have a lot of overlap when the predicted probability is in the range of 0 to 0.6, suggesting that classification is a very challenging job given the variables used in this study. For example, using the threshold value of 0.5 (θ = 0.5; this value is commonly used when data is balanced), we can divide the figure into two parts. On the right of notation “θ = 0.5” in the figure, the area under the red curve is the size of true positives, and the area under the blue curve is the size of false positives. On the left of notation “θ = 0.5,” the area under the red curve is the size of false negatives, and the area under the blue curve is the size of true negatives. Table 3C summarizes the relative size of these four areas using confusion matrix. The computation results showed that while the accuracy rate was as high as 91.10%, the recall rate was at a very low level of 5.54% (the choice of such threshold value yielded a very low detection rate of MDESI cases); this is a common phenomenon when the data are unbalanced. False identifications (false positives & false negatives) also vary when the threshold line shifts.

**Figure 4:**
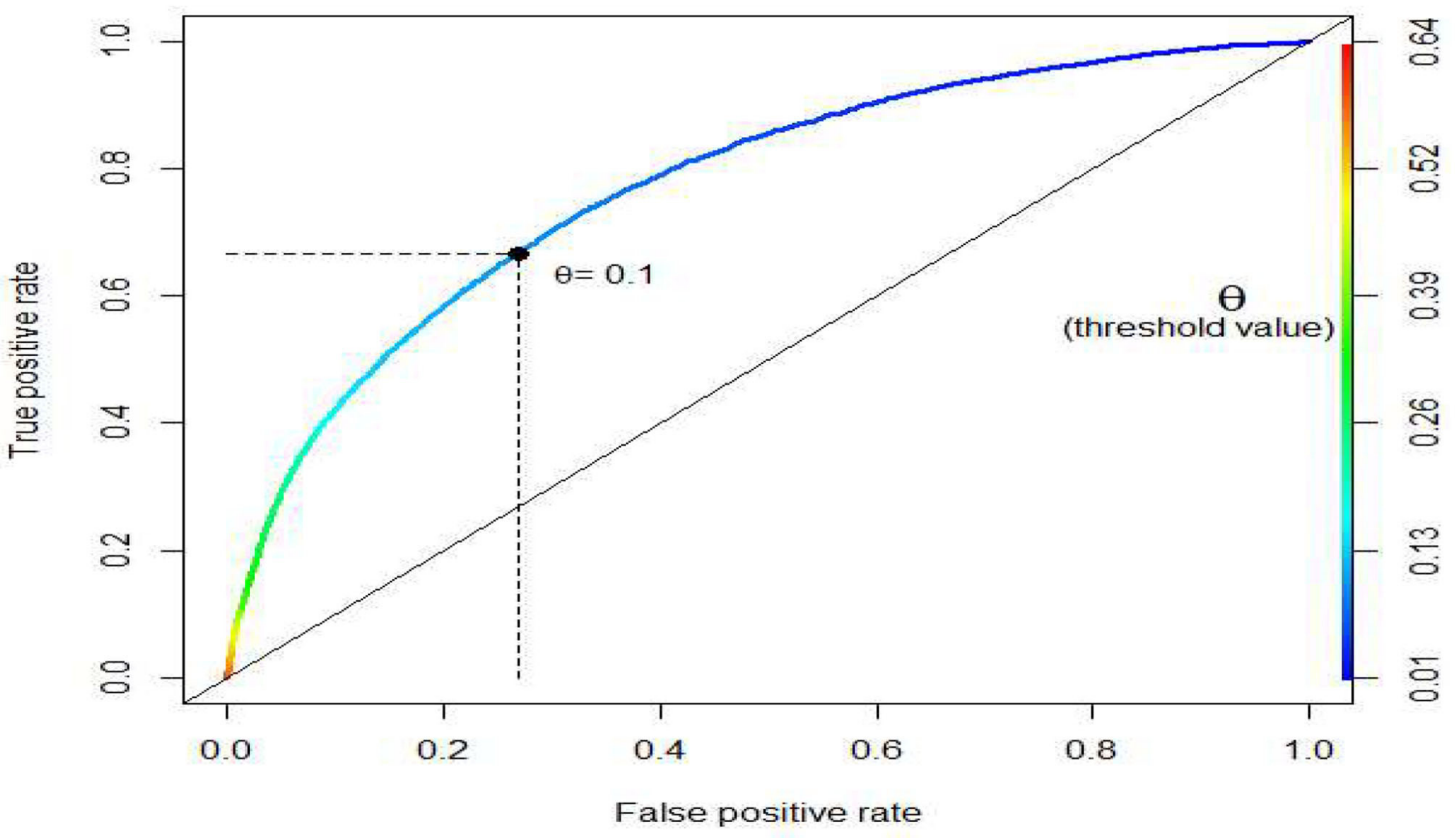
Receiver Operating Characteristic (ROC) curve.

Using the threshold value of 0.1 instead of 0.5, a better recall rate of 66.78% was obtained at the expense of a lower accuracy rate of 72.48%. This is a worthy trade-off since for every one-unit decrease in accuracy rate, the recall rate increases by about 3.3 units ((66.78–5.54)/(91.10–72.48) ≈ 3.3). The receiver operating characteristic (ROC) curve, depicting the relationship of true positives and false positives, is plotted in Figure 4. We used ROC to show another (marginal) trade-off between true positives and false positives as the threshold value θ decreased. The optimal threshold value (θ = 0.1) is also shown Figure 4 for reference purpose. Notably, a more precise optimal threshold value can be found when these two curves in Figure 2 intersect with each other. At the intersection point, the threshold value θ is 0.095, and 70% of accuracy and recall rate can be obtained. The approximate optimal value of 0.1 was taken since the precise value could vary slightly, due to the random data splitting process.

One of the important lessons learned in this research project is that machine learning methods can be used as a complementary tool for traditional inferential statistical methods. For example, besides making inferences about the estimated coefficient vector β in the logistic regression model, we were able to use the predicted probability and a selection of an optimal threshold value θ to build a classifier. Admittedly, there is lot of room for improvement in our predictive model. First, the McFadden's pseudo R-squared (23, 24) in the logistic regression results—as shown at the bottom of Table 2—was 14.22%, suggesting the existence of other essential features that were missing in the model. While the inclusion of independent variables is subject to data availability, the possible interactions of independent variables needs to be examined and included if they improve the model's explanatory power. Secondly, besides using recall and accuracy to choose an optimal threshold value θ, other performance metrics such as F1-score can be considered and used to deal with imbalanced data problem. The choice of model performance metrics depends on the cost of false identifications. We deemed it critical to identify more MDESI cases, and therefore, recall was used besides accuracy to reduce false negatives. Finally, there are other machine learning techniques that we can adopt if the only goal is to build a powerful predictive model without worrying about the use of inferential statistics. Supervised machine learning tools such as support vector machine, decision trees, Naïve Bayes, etc. can be applied to the NSDUH data in the future projects.

## Conclusion

Major depression in adolescent is a serious public health problem. Effective interventions require the identification of those who are prone to, or likely have undiagnosed cases of, MDESI. Our empirical findings indicate that sociodemographic factors such as gender, age, race/ethnicity, family composition, parenting style, and school experiences are significantly associated with severe functional impairment among adolescents with major depression. As the large effect sizes point out, girls and adolescents with bad school experiences are particularly susceptible to being severely impaired by major depression. More gender-specific and school mental health programs are warranted to prevent the onset of depression among these two groups. To put our preliminary predictive model into work, risk scores can be computed for evaluating and detecting the potential MDESI cases in adolescents. There is still much room for improvement if a more functional predictive model is needed. Several weaknesses need to be addressed and resolved in the next research project. First, the dependent variable in our predictive model includes both current and 12-month MDESI cases based on how the variable was defined in the NSDUH. Therefore, the model could not be used to differentiate past MDESI cases from the current cases. Second, the temporal relationship and direction of association between MDESI and covariates cannot be easily discerned by using cross-sectional survey data. Thirdly, more potential covariates need to be considered in addition to the sociodemographic factors, such as substance use disorders that often co-occur with depression during adolescence. Future studies using machine learning techniques can build upon the predictive model that we introduced in this study to help us understand better predictors of major depression in adolescents from a social science perspective.

## Data Availability Statement

Publicly available datasets were analyzed in this study. This data can be found here: https://www.datafiles.samhsa.gov/study-series/national-survey-drug-use-and-health-nsduh-nid13517.

## Ethics Statement

The studies involving human participants were reviewed and approved by The Institutional Review Board at RTI International approved the NSDUH data collection protocol. Written informed consent to participate in this study was provided by the participants' legal guardian/next of kin.

## Author Contributions

I-MC conceptualized the research question, conducted data analysis, and drafted the manuscript. WL, FT, and DH participated in drafting and revising the manuscript and conducted literature review that informed the introduction and discussion. All authors approved the final version of this manuscript.

## Conflict of Interest

The authors declare that the research was conducted in the absence of any commercial or financial relationships that could be construed as a potential conflict of interest.
